# Association of inflammatory indicators with intensive care unit mortality in critically ill patients with coronary heart disease

**DOI:** 10.3389/fimmu.2023.1295377

**Published:** 2023-11-14

**Authors:** Yuan Cheng, Yang Chen, Mengxia Mao, Ruixuan Wang, Jun Zhu, Qing He

**Affiliations:** ^1^Key Laboratory of Advanced Technologies of Materials, Ministry of Education, School of Materials Science and Engineering, Southwest Jiaotong University, Chengdu, Sichuan, China; ^2^Liverpool Centre for Cardiovascular Science at University of Liverpool, Liverpool John Moores University and Liverpool Heart & Chest Hospital, Liverpool, United Kingdom; ^3^The First Affiliated Hospital of Yangtze University, Jingzhou, Hubei, China; ^4^School of Electronics and Computer Science, University of Liverpool, Liverpool, United Kingdom; ^5^School of Civil Engineering, Southwest Jiaotong University, Chengdu, Sichuan, China; ^6^Department of Intensive Care Medicine, Affiliated Hospital of Southwest Jiaotong University/The Third People’s Hospital of Chengdu, Chengdu, Sichuan, China

**Keywords:** inflammatory indicator, coronary heart disease, intensive care unit, MIMIC, eICU, mortality, nomogram

## Abstract

**Objective:**

Coronary heart disease (CHD) is one of the major cardiovascular diseases, a common chronic disease in the elderly and a major cause of disability and death in the world. Currently, intensive care unit (ICU) patients have a high probability of concomitant coronary artery disease, and the mortality of this category of patients in the ICU is receiving increasing attention. Therefore, the aim of this study was to verify whether the composite inflammatory indicators are significantly associated with ICU mortality in ICU patients with CHD and to develop a simple personalized prediction model.

**Method:**

7115 patients from the Multi-Parameter Intelligent Monitoring in Intensive Care Database IV were randomly assigned to the training cohort (n = 5692) and internal validation cohort (n = 1423), and 701 patients from the eICU Collaborative Research Database served as the external validation cohort. The association between various inflammatory indicators and ICU mortality was determined by multivariate Logistic regression analysis and Cox proportional hazards model. Subsequently, a novel predictive model for mortality in ICU patients with CHD was developed in the training cohort and performance was evaluated in the internal and external validation cohorts.

**Results:**

Various inflammatory indicators were demonstrated to be significantly associated with ICU mortality, 30-day ICU mortality, and 90-day ICU mortality in ICU patients with CHD by Logistic regression analysis and Cox proportional hazards model. The area under the curve of the novel predictive model for ICU mortality in ICU patients with CHD was 0.885 for the internal validation cohort and 0.726 for the external validation cohort. The calibration curve showed that the predicted probabilities of the model matched the actual observed probabilities. Furthermore, the decision curve analysis showed that the novel prediction model had a high net clinical benefit.

**Conclusion:**

In ICU patients with CHD, various inflammatory indicators were independent risk factors for ICU mortality. We constructed a novel predictive model of ICU mortality risk in ICU patients with CHD that had great potential to guide clinical decision-making.

## Introduction

Coronary heart disease (CHD), one of the major cardiovascular diseases, is a common chronic disease in the elderly and is the leading cause of disability and death in the world (1). Moreover, due to population growth and ageing, the economic consequences of this chronic disease affect many levels of society and will be an increasing burden, especially for low- and middle-income countries (2). The Prospective Urban and Rural Epidemiology study shows that secondary prevention of cardiovascular disease remains inaccessible and unaffordable for most communities and households in upper middle-income, lower middle-income and low-income countries (3). Patients with CHD are recently receiving increased attention in the ICU, and a dedicated arrhythmia monitoring and treatment unit, the coronary ICU, has emerged to care for ICU patients with CHD. ICU patients with CHD require more life support and have more complications (*e.g.* central venous catheter infections, ventilator-associated pneumonia, etc.), which have led to a significant increase in ICU mortality, length of ICU stay, and healthcare costs for ICU patients with CHD (4). Therefore, the prognostic management of ICU patients with CHD is of great importance.

The main known risk factors for the diagnosis or prognosis of CHD include dyslipidemia, high blood pressure, diabetes, and smoking (5). Also, obesity and the metabolic syndrome would lead to an increased risk of CHD (6), possibly because insulin resistance is a major feature of the metabolic syndrome. Inflammation plays a key role in the development and progression of atherosclerosis, as indicated by a unified view of the pathophysiology of atherosclerosis (7), and inflammation contributes to an increased risk of cardiovascular events (8). Inflammatory signaling alters the behavior of endothelial cells and smooth muscle and recruits more interacting inflammatory cells to promote lesion formation and complications (9). Li et al. reported that cytoleukin-6, C-reactive protein (CRP), complement, CD40 and myeloperoxidase could be used to assess the severity of CHD (10). Overall, inflammatory indicators have great potential as prognostic predictors in ICU patients with CHD.

Blood test is widely used as a simple and inexpensive test for various diseases. Previous studies have shown the predictive value of inflammatory indicators for all-cause mortality in cardiovascular disease. For example, in patients with non-ST-segment elevation myocardial infarction and ST-segment elevation myocardial infarction, the platelet-lymphocyte ratio (PLR) ratio is an independent predictor for mortality (11). Similarly, Osadnik et al. found that higher mortality in patients with stable coronary artery disease who underwent stenting in patients with the highest PLR values (12). Other study indicated that neutrophil-lymphocyte ratio (NLR) had potential to be an independent prognostic factor in CHD patients (13). Moreover, Xiao et al. reported that a U-shaped association between the systemic inflammatory index (SII) and all-cause mortality in patients with cardiovascular disease in the general individuals, which could be used as a clinical predictor (14). Xia et al. revealed that the systemic inflammatory response index (SIRI) was significantly associated with myocardial infarction in patients < 60 years, but not SII (15). In addition, red blood cell distribution width (RDW) is a useful tool for differentiating between inflammatory and non-inflammatory joint disease in clinical practice (16). Hou et al. found RDW to be an independent risk factor for frailty in elderly patients with coronary artery disease (17). However, there is a lack of studies on the correlation between inflammatory markers and mortality in ICU patients with CHD.

The aims of our study were: (1) determining the association of several inflammatory indicators with all-cause ICU mortality in ICU patients with CHD, including SII, SIRI, NLR, PLR, neutrophil to lymphocyte platelet ratio (NLPR), aggregate index of systemic inflammation (AISI), and RDW; (2) constructing a novel model based on these indicators and severity score to predict ICU mortality in ICU patients with CHD.

## Methods

### Sources of data

Our study data were obtained from a publicly accessible Multi-Parameter Intelligent Monitoring in Intensive Care Database IV (MIMIC IV, version 2.0, recruitment during 2012 to 2019) as detailed in previous publications (18). In addition, we extracted external validation cohort from the eICU Collaborative Research Database (EICU, version 2.0, recruitment during 2014 to 2015) (19). As all data were anonymous, the patient consent was irrelevant. All data in this study were extracted by the author Yang Chen, who obtained access to the database and relevant credentials (NO. 36328122).

### Study participants

**Figure 1:**
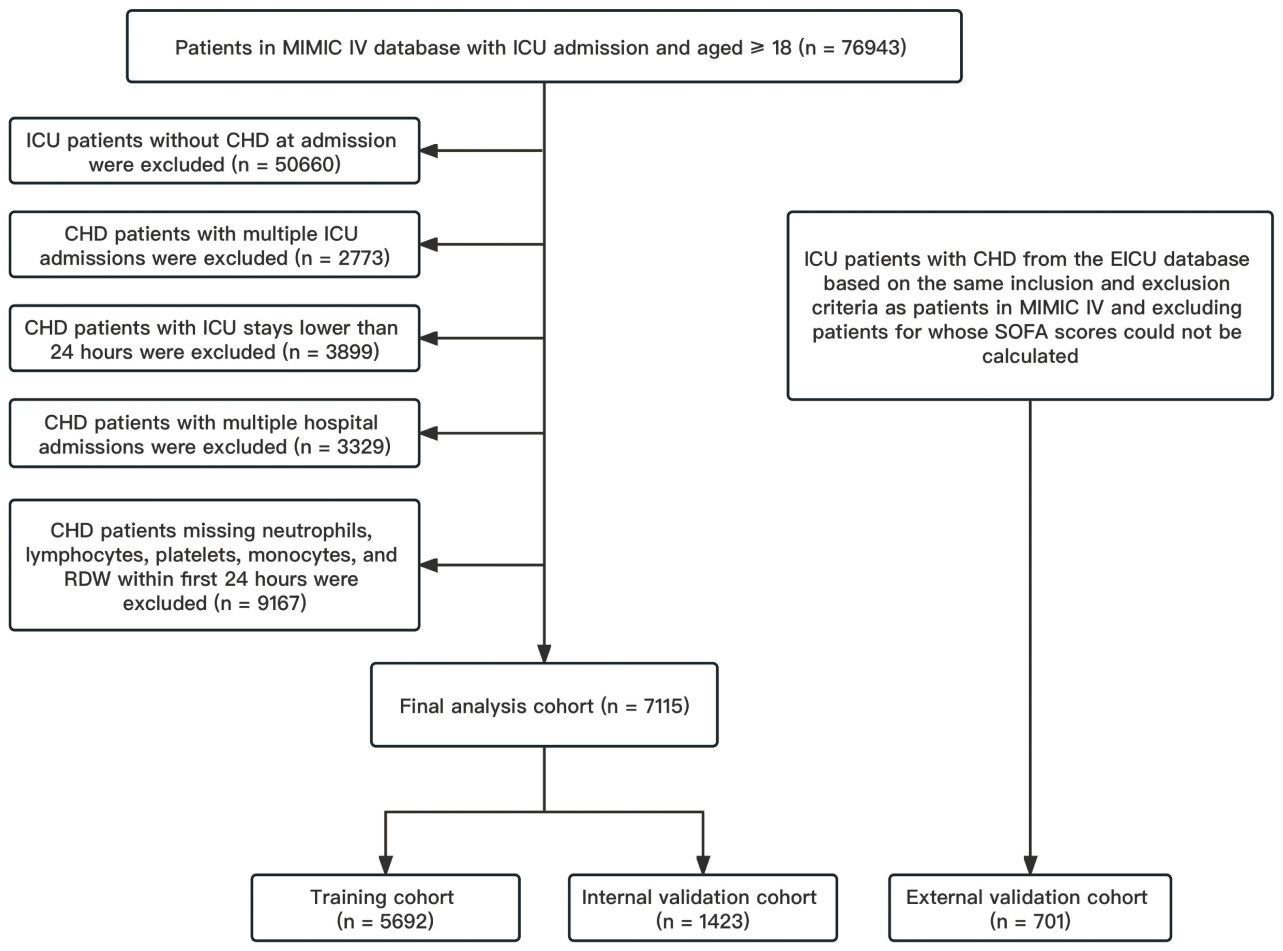
Flow chart of patient screening. CHD, coronary heart disease; EICU, eICU Collaborative Research Database; ICU, intensive care unit; MIMIC IV, Multi-Parameter Intelligent Monitoring in Intensive Care Database IV; SOFA, sequential organ failure assessment.

Our study included all adult patients with CHD admitted to the ICU according to International Classification of Diseases (ICD) version 9 or 10 (We included all patients who contained any ICD codes related to coronary artery). The exclusion criteria were as follows (1) records of multiple ICU admissions other than the first ICU admission; (2) records of ICU stays of less than 24 hours; (3) records of repeated multiple hospitalizations; (4) exclusion of records of missing neutrophils, lymphocytes, monocytes, platelets, and RDW. Finally, a total of 7115 patients were extracted from MIMIC IV for initial analysis and model construction, and 701 patients were extracted from EICU for external validation (details shown in **Figure 1**).

### Extraction of variables and study outcomes

We extracted the following data: demographic characteristics, vital signs, comorbidities, severity scores on admission to the ICU, laboratory results (within the first day of admission to the ICU), interventions, and medications. For variables measured multiple times, we used the first value. Our primary outcomes: all-cause mortality during ICU admission (ICU mortality); Secondary outcome: all-cause 30-day mortality after ICU admission (30-day ICU mortality) and all-cause 90-day mortality after ICU admission (90-day ICU mortality).

### Definition

SII was defined as platelet × neutrophil/lymphocyte. SIRI was defined as neutrophil × monocyte/lymphocyte. NLP was defined as neutrophil/lymphocyte. PLR was defined as platelet/lymphocyte. NLPR was defined as neutrophil/(lymphocyte × platelet). AISI was defined as neutrophils × monocytes × platelets/lymphocytes.

### Statistical analysis

For continuous variables, normality was tested using the Shapiro-Wilk test. And depending on the type and distribution of the variable, the normal distribution was expressed as mean and standard deviation (SD) and differences between groups were assessed using the t-test, and the non-normal distribution was expressed as median and interquartile range (IQR) and differences between groups were assessed using the Kruskal-Wallis test. For categorical variables, expressed as counts and percentages, differences between groups were assessed using the Chi^2^ or Fisher’s exact test.

We analyzed the cumulative rate of all-cause mortality in CHD patients within 90 days of ICU admission using Kaplan-Meier survival analysis to compare the cumulative distribution of deaths among patients in four-score subgroups for each indicator at admission. We also used the restricted cubic spline function (RCS) to explore the nonlinear relationship between these metrics and the study outcomes when used as continuous variables.

To further assess the independent associations between the indicators and the primary endpoints, we used Logistic regression model and Cox proportional hazards model, and we used different models to adjust for potential confounders. Model 1: crude analysis without adjusting for any confounders; Model 2: adjustments including age, male, and race; Model 3: additional adjusting for confounders (heart rate, respiratory rate, saturation of peripheral oxygen, sequential organ failure assessment (SOFA), acute exacerbation of chronic obstructive pulmonary disease, congestive heart failure, malignant cancer, dyslipidemia, acute respiratory failure, acute kidney failure, potassium, aniongap, blood urea nitrogen, glucose, serum creatinine, hematocrit, hemoglobin, mean corpuscular volume, red blood cell, dialysis, vasopressor, invasive mechanical ventilation, coronary artery bypass graft, angiotensin-converting enzyme inhibitors or angiotensin receptor blocker, antiplatelet, statin, non-vitamin K antagonist oral anticoagulant, and Vitamin K antagonists) based on Model 2. Once the indicators were grouped based on quartiles, we chose G1 as the reference group and calculated the adjusted odds ratio (OR) or hazard ratio (HR) of the primary endpoints for the other groups in comparison to the reference group.

Given the importance of mortality risk management in ICU patients with CHD, then we used these inflammatory indictors in conjunction with the widely utilized SOFA to construct a novel predictive model of ICU mortality in ICU patients with CHD. We first divided these inflammatory indicators into elevated value and non-elevated value groups based on their respective third quartiles. Subsequently, we divided the entire cohort into a training cohort and an internal validation cohort on a 8:2 basis. In the training corhort, we used univariate Logistic regression analyses followed by stepwise forward multivariate Logistic regression analyses to select the variables used to construct the novel predictive model, computed correlation coefficient and variance inflation factor (VIF) to detect covariance of the variables in the model, and used the Hosmer-Lemeshow test to assess the fit of the logistic regression models. Then, the area under the curve (AUC) of receiver operating characteristic (ROC) curves, plotting of calibration curves, decision curve analysis (DCA) (compared with SOFA) were used in both the internal and external validation cohorts, in addition, we computed the integrated discrimination improvement (IDI) to validate the variability of the predictive performance of the new model between the novel model and SOFA. Furthermore, we transformed the novel model obtained from the training cohort to nomogram and interactive network dynamic nomogram.

We performed all statistical processing using SPSS (version 29), Stata (version 17), and R (version 4.2.3). In all analyses, a two-tailed *P* < 0.05 was considered statistically significant.

## Results

### Comparison of ICU survival and death in ICU patients with CHD

In our study, 7115 ICU patients with CHD were included and the median (IQR) age of all patients was 71.43 (63.08 - 79.60), 68.9% patients were male and 69.7% patients were White. We divided all patients into two groups based on ICU survival and death (details shown in **Table 1**), and we found that the ICU death group was significantly older than the survival group (median 75.25, IQR [65.65 - 82.34] vs. median 71.19, IQR [71.19 - 79.14], *P* < 0.001), and that the ICU death group had fewer males, lower White individuals, and more comorbidities. In addition, the ICU death group had higher value of indicators than the survivor group.

**Table 1:** Baseline characteristics of ICU patients with CHD.

Characterisitics	All	ICU survival	ICU death	*P-value*
N	7115	6553	562	
Age, years	71.43 (63.08, 79.60)	71.19 (62.91, 79.14)	75.25 (65.65, 82.34)	< 0.001
Male, n (%)	4900 (68.9)	4566 (69.7)	334 (59.4)	< 0.001
Race, n (%)				< 0.001
White	4961 (69.7)	4616 (70.4)	345 (61.4)	
Non-white	2154 (30.3)	1937 (29.6)	217 (38.6)	
BMI, kg/m^2^	28.13 (24.60, 32.42)	28.15 (24.69, 32.39)	27.60 (23.71, 32.59)	0.099
Vital sign				
Heart rate	81.00 (74.00, 92.00)	80.00 (74.00, 91.00)	92.00 (79.00, 107.00)	< 0.001
Mean blood pressure	78.00 (69.00, 89.00)	78.00 (69.00, 88.00)	78.00, (66.00, 90.00)	0.292
Respiratory rate	16.00 (15.00, 21.00)	16.00 (14.50, 20.00)	21.00 (18.00, 26.00)	< 0.001
Temperature	36.67 (36.43, 36.94)	36.67 (36.44, 36.94)	26.69 (36.39, 37.06)	0.107
SpO_2_	99.00 (96.00, 100.00)	99.00 (96.00, 100.00)	97.00 (93.00, 100.00)	< 0.001
SOFA score	5 (3.8)	5 (3.8)	11 (8.14)	< 0.001
Comorbidities, n (%)				
AECOPD	198 (2.8)	173 (2.6)	25 (4.4)	0.012
AF	2863 (40.2)	2619 (40.0)	244 (43.4)	0.109
CHF	2893 (40.7)	2595 (39.6)	298 (53.0)	< 0.001
Diabetes	2929 (41.2)	2693 (41.4)	236 (42.0)	0.678
Malignant cancer	591 (8.3)	504 (7.7)	87 (15.5)	< 0.001
Dyslipidemia	4622 (65.0)	4381 (66.9)	241 (42.9)	< 0.001
ARF	1352 (19.0)	1035 (15.8)	317 (56.4)	< 0.001
AKF	2374 (33.4)	1989 (30.4)	385 (68.5)	< 0.001
Malignant hypertension	49 (0.7)	48 (0.7)	1 (0.2)	0.127
Laboratory				
Lymphocytes, ×10^3^/μL	1.41 (0.87, 2.12)	1.46 (0.90, 2.17)	0.92 (0.52, 1.46)	< 0.001
Monocytes, ×10^3^/μL	0.48 (0.29, 0.77)	0.47 (0.29, 0.75)	0.64 (0.34, 1.05)	< 0.001
Neutrophils, ×10^3^/μL	9.54 (6.61, 13.18)	9.42 (6.59, 12.89)	11.69 (7.25, 17.40)	< 0.001
Platelets, ×10^3^/μL	162.00 (121.00, 220.00)	161.00 (122.00, 217.00)	179.00 (119.00, 261.00)	< 0.001
Sodium, mmol/L	139.00 (136.00, 141.00)	139.00 (136.00, 141.00)	138.00 (135.00, 142.00)	0.511
Potassium, mmol/L	4.30 (3.90, 4.70)	4.30 (3.90, 4.70)	4.40 (3.80, 4.90)	0.018
Aniongap, %	13.00 (11.00, 16.00)	13.00 (11.00, 16.00)	17.00 (14.00, 20.00)	< 0.001
BUN, mg/dL	20.00 (14.00, 31.00)	19.00 (14.00, 29.00)	63.50 (21.00, 55.00)	< 0.001
Glucose, mg/dL	125.00 (106.00, 157.00)	124.00 (106.00, 153.00)	155.50 (116.00, 213.00)	< 0.001
Creatinine, mg/dL	1.00 (0.80, 1.50)	1.00 (0.80, 1.40)	1.50 (1.10, 2.50)	< 0.001
Hematocrit, g/dL	29.90 (26.00, 34.50)	29.70 (25.90, 34.30)	31.90 (27.40, 36.60)	< 0.001
Hemoglobin, g/dL	9.90 (8.5, 11.40)	9.90 (8.50, 11.40)	10.30 (8.90, 11.90)	< 0.001
MCV, fl	92.00 (88.00, 95.00)	91.00 (88.00, 95.00)	93.00 (88.00, 98.00)	< 0.001
Red blood cell, m/μL	3.29 (2.82, 3.81)	3.27 (2.83, 3.79)	3.45 (2.93, 4.05)	< 0.001
MCH, pg	30.40 (28.90, 31.60)	30.40 (29,00, 31.60)	30.30 (28.60, 31.60)	0.177
Interventions, n (%)				
Dialysis	301 (4.2)	218 (3.3)	83 (14.8)	< 0.001
Vasopressor	4115 (57.8)	3700 (56.5)	415 (73.8)	< 0.001
IMV	3750 (52.7)	3362 (51.3)	388 (69.0)	< 0.001
CABG	3088 (43.4)	3055 (46.6)	33 (5.9)	< 0.001
PCI	391 (5.5)	356 (5.4)	35 (6.2)	0.440
Medication, n (%)				
ACEI/ARB	2228 (31.3)	2177 (33.2)	51 (9.1)	< 0.001
Antiplatelet	6132 (86.2)	5773 (88.1)	359 (63.9)	< 0.001
Statin	5352 (75.2)	5066 (77.3)	286 (50.9)	< 0.001
Beta blocker	526 (7.4)	485 (7.4)	41 (7.3)	0.927
NOAC	418 (5.9)	405 (6.2)	13 (2.3)	< 0.001
VKA	1691 (23.8)	1638 (25.0)	53 (9.4)	< 0.001
Inflammation indicators				
SII	1022.74 (548.96, 2167.45)	962.74 (535.09, 1997.31)	2286.03 (1035.45, 4781,67)	< 0.001
SIRI	3.17 (1.41, 6.99)	2.97 (1.35, 5.35)	7.72 (3.38, 17.70)	< 0.001
NLR	6.35 (3.93, 11.33)	6.08 (3.81, 10.94)	12.24 (7.18, 22.15)	< 0.001
PLR	113.45 (66.67, 217.92)	108.89 (65.56, 204.40)	207.68 (109.34, 361.11)	< 0.001
NLPR	0.04 (0.02, 0.07)	0.04 (0.02, 0.07)	0.07 (0.04, 0.14)	< 0.001
AISI	518.40 (196.08, 1349.60)	418.52 (187.88, 1202.65)	1472.86 (480.47, 3584.00)	< 0.001
RDW, %	14.00 (13.20, 15.50)	13.90 (13.10, 15.30)	15.50 (14.10, 17.30)	< 0.001

ACEI, angiotensin-converting enzyme inhibitors; AECOPD, acute exacerbation of chronic obstructive pulmonary disease; AF, atrial fibrillation; AISI, aggregate index of systemic inflammation; AKF, acute kidney failure; ARB, angiotensin receptor blocker; ARF, acute respiratory failure; BMI, body mass index; BUN, blood urea nitrogen; CABG, coronary artery bypass graft; CHF, congestive heart failure; IMV, invasive mechanical ventilation; MCH, mean corpsular hemoglobin; MCV, mean corpuscular volume; NLPR, neutrophil to lymphocyte platelet ratio; NLR, neutrophil-lymphocyte ratio; NOAC, non-vitamin K antagonist oral anticoagulant; PCI, percutaneous coronary intervention; PLR, platelet-lymphocyte ratio; RDW, red blood cell volume distribution width; SII, systemic inflammatory index; SIRI, systemic inflammatory response index; SOFA, sequential organ failure assessment; SpO_2_, saturation of peripheral oxygen; VKA, Vitamin K antagonists.

### Association between inflammatory indicators and ICU mortality in ICU patients with CHD

**Figure 2:**
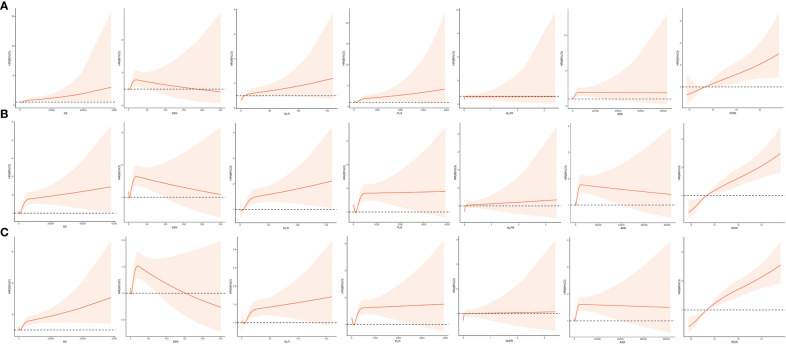
Restricted cubic spline function between inflammation indicators (SII, SIRI, NLR, PLR, NLPR, AISI, RDW) and ICU mortality **(A)**, ICU 30-day mortality **(B)**, and ICU 90-day mortality **(C)**. AISI, aggregate index of systemic inflammation; NLPR, neutrophil to lymphocyte platelet ratio; NLR, neutrophil-lymphocyte ratio; PLR, platelet-lymphocyte ratio; RDW, red blood cell volume distribution width; SII, systemic inflammatory index; SIRI, systemic inflammatory response index.

Based on **Table 2**, the results of multivariate logistic regression models after adjusting for baseline characteristics, vital signs, comorbidities, laboratory results, interventions, and medications showed the elevated inflammatory indicators, except for the NLPR, were independent risk factors for ICU mortality in ICU patients with CHD, among which the highest risk was for RDW (first quartile as reference group; fourth quartile: OR = 2.05, 95% CI: 1.35 - 3.10, *P* < 0.001) and AISI (first quartile as reference group; fourth quartile: OR = 1.68, 95% CI: 1.19 - 2.36, *P* = 0.003). Furthermore, the association of each inflammation indicator with ICU mortality when used as a continuous variable was shown in **Figure 2A**, which implied that the greater the values of SII, NLR, PLR, AISI, and RDW, the higher ICU mortality of ICU patients with CHD.

**Table 2:** The association of each inflammatory indicator with ICU mortality in ICU patients with CHD.

Categories	Model 1			Model 2			Model 3		
	OR (95% CI)	*P*-value	*P* for trend	OR (95% CI)	*P*-value	*P* for trend	OR (95% CI)	*P*-value	*P* for trend
SII (Quartile^a^)									
Q1 (N = 1778)	Ref.		< 0.001	Ref.		< 0.001	Ref.		< 0.001
Q2 (N = 1780)	0.65 (0.46, 0.92)	0.015		0.67 (0.47, 0.95)	0.023		0.77 (0.50, 1.18)	0.228	
Q3 (N = 1779)	1.67 (1.25, 2.22)	< 0.001		1.71 (1.28, 2.27)	< 0.001		1.25 (0.88, 1.79)	0.220	
Q4 (N = 1778)	4.08 (3.16, 5.26)	< 0.001		3.93 (3.04, 5.08)	< 0.001		1.61 (1.15, 2.26)	0.005	
SIRI (Quartile^a^)									
Q1 (N = 1776)	Ref.		< 0.001	Ref.		< 0.001	Ref.		0.043
Q2 (N = 1782)	1.37 (0.96, 1.95)	0.080		1.39 (0.98, 1.98)	0.068		1.13 (0.75, 1.71)	0.557	
Q3 (N = 1779)	2.36 (1.71, 3.26)	< 0.001		2.38 (1.72, 3.28)	< 0.001		1.24 (0.85, 1.81)	0.274	
Q4 (N = 1778)	6.31 (4.71, 8.46)	< 0.001		6.12 (4.56, 8.21)	< 0.001		1.56 (1.09, 2.23)	0.016	
NLR (Quartile^a^)									
Q1 (N = 1774)	Ref.		< 0.001	Ref.		< 0.001	Ref.		0.012
Q2 (N = 1781)	0.98 (0.69, 1.44)	0.996		1.03 (0.71, 1.48)	0.887		0.93 (0.61, 1.42)	0.732	
Q3 (N = 1783)	2.50 (1.83, 3.41)	< 0.001		2.54 (1.86, 3.47)	< 0.001		1.47 (1.02, 2.13)	0.039	
Q4 (N = 1777)	5.96 (4.48, 7.97)	< 0.001		5.88 (4.40, 7.85)	< 0.001		1.49 (1.05, 2.12)	0.025	
PLR (Quartile^a^)									
Q1 (N = 1783)	Ref.		< 0.001	Ref.		< 0.001	Ref.		< 0.001
Q2 (N = 1775)	0.78 (0.57, 1.09)	0.144		0.79 (0.57, 1.09)	0.150		0.79 (0.53, 1.19)	0.255	
Q3 (N = 1779)	1.68 (1.27, 2.22)	< 0.001		1.61 (1.22, 2.13)	< 0.001		0.90 (0.62, 1.29)	0.556	
Q4 (N = 1778)	3.61 (2.80, 4.65)	< 0.001		3.40 (2.63, 4.39)	< 0.001		1.50 (1.06, 2.12)	0.023	
NLPR (Quartile^a^)									
Q1 (N = 1784)	Ref.		< 0.001	Ref.		< 0.001	Ref.		0.124
Q2 (N = 1807)	1.19 (0.86, 1.64)	0.291		0.23 (0.18, 0.31)	< 0.001		1.03 (0.71, 1.49)	0.895	
Q3 (N = 1724)	1.94 (1.44, 2.61)	< 0.001		0.29 (0.22, 0.37)	< 0.001		1.42 (1.01, 2.01)	0.046	
Q4 (N = 1800)	4.27 (3.26, 5.59)	< 0.001		0.46 (0.37, 0.58)	< 0.001		1.10 (0.80, 1.53)	0.554	
AISI (Quartile^a^)									
Q1 (N = 1779)	Ref.		< 0.001	Ref.		< 0.001	Ref.		< 0.001
Q2 (N = 1778)	1.19 (0.86, 1.64)	0.291		1.03 (0.74, 1.44)	0.842		1.14 (0.77, 1.70)	0.511	
Q3 (N = 1780)	1.94 (1.44, 2.61)	< 0.001		1.67 (1.18, 2.16)	0.002		1.03 (0.70, 1.49)	0.898	
Q4 (N = 1778)	4.27 (3.26, 5.59)	< 0.001		4.45 (3.41, 5.80)	< 0.001		1.68 (1.19, 2.36)	0.003	
RDW (Quartile^a^)									
Q1 (N = 2044)	Ref.		< 0.001	Ref.		< 0.001	Ref.		0.002
Q2 (N = 1365)	2.47 (1.72, 3.55)	< 0.001		2.35 (1.63, 3.39)	< 0.001		1.44 (0.94, 2.21)	0.096	
Q3 (N = 1876)	3.51 (2.53, 4.88)	< 0.001		3.20 (2.29, 4.47)	< 0.001		1.39 (0.93, 2.07)	0.112	
Q4 (N = 1830)	7.54 (5.53, 10.29)	< 0.001		6.91 (5.05, 9.46)	< 0.001		2.05 (1.35, 3.10)	< 0.001	

Model 1: unadjusted; Model 2: adjusted for age, male, race; Model 3: adjusted for age, male, race, heart rate, respiratory rate, saturation of peripheral oxygen, sequential organ failure assessment score, acute exacerbation of chronic obstructive pulmonary disease, congestive heart failure, malignant cancer, dyslipidemia, acute respiratory failure, acute kidney failure, potassium, aniongap, blood urea nitrogen, glucose, serum creatinine, hematocrit, hemoglobin, mean corpuscular volume, red blood cell, dialysis, vasopressor, invasive mechanical ventilation, coronary artery bypass graft, angiotensin-converting enzyme inhibitors or angiotensin receptor blocker, antiplatelet, statin, non-vitamin K antagonist oral anticoagulant, and Vitamin K antagonists.

a RDW categries: Q1 (x < 13.20), Q2 (13.20 ≤ x < 14.00), Q3 (14.00 ≤ x < 15.50), Q4 (x ≥ 15.50); SII categries: Q1 (x < 548.96), Q2 (548.96 ≤ x < 1022.74),Q3 (1022.74 ≤ x < 2167.45), Q4 (x ≥ 2167.45); SIRI categries: Q1 (x < 1.41), Q2 (1.41 ≤ x < 3.17), Q3 (3.17 ≤ x < 6.99), Q4 (x ≥ 6.99); NLR categries: Q1 (x < 3.93), Q2 (3.93 ≤ x < 6.35), Q3 (6.35 ≤ x < 11.38), Q4 (x ≥ 11.35); PLR categries: Q1 (x < 66.67), Q2 (66.67 ≤ x < 113.48), Q3 (113.48 ≤ x < 217.92), Q4 (x ≥ 217.92); NLPR categries: Q1 (x < 0.02), Q2 (0.03 ≤ x < 0.04), Q3 (0.04 ≤ x < 0.68), Q4 (x ≥ 0.68); AISI categries: Q1 (x < 196.08), Q2 (196.08 ≤ x < 518.40), Q3 (518.40 ≤ x < 1349.60), Q4 (x ≥ 1349.60).

AISI, aggregate index of systemic inflammation; CI, confidence interval; NLPR, neutrophil to lymphocyte platelet ratio; NLR, neutrophil-lymphocyte ratio; OR, odds ratio; PLR, platelet-lymphocyte ratio; RDW, red blood cell volume distribution width; SII, systemic inflammatory index; SIRI, systemic inflammatory response index.

### Association between inflammatory indicators and 30-day ICU mortality in ICU patients with CHD

Based on **Table 3**, the results of multivariate Cox proportional hazards models after adjusting for baseline characteristics, vital signs, comorbidities, laboratory results, interventions, and medications showed all the elevated inflammatory indicators were independent risk factors for ICU mortality in ICU patients with CHD, among which the highest risk was for RDW (first quartile as reference group; second quartile: HR = 1.62, 95% CI: 1.13 - 2.32, *P* = 0.009; third quartile: HR = 1.80, 95% CI: 1.30 - 2.52, *P* < 0.001; fourth quartile: HR = 2.96, 95% CI: 2.10 - 4.17, *P* < 0.001) and AISI (first quartile as reference group; fourth quartile: HR = 1.80, 95% CI: 1.37 - 2.37, *P* < 0.001). Moreover, the association of each inflammation indicator with 30-day ICU mortality when used as a continuous variable was shown in **Figure 2B**, which implied that the greater the values of SII, NLR, PLR, NLPR, and RDW, the higher 30-day ICU mortality of ICU patients with CHD.

**Table 3:** The association of each inflammatory indicator with 30-day ICU mortality in ICU patients with CHD.

Categories	Model 1			Model 2			Model 3		
	HR (95% CI)	*P*-value	*P* for trend	HR (95% CI)	*P*-value	*P* for trend	HR (95% CI)	*P*-value	*P* for trend
SII (Quartile^a^)									
Q1 (N = 1778	Ref.		< 0.001	Ref.		< 0.001	Ref.		< 0.001
Q2 (N = 1780)	0.74 (0.57, 0.97)	0.028		0.77 (0.59, 1.01)	0.057		0.95 (0.73, 1.24)	0.722	
Q3 (N = 1779)	1.50 (1.19, 1.89)	< 0.001		1.55 (1.23, 1.95)	< 0.001		1.05 (0.83, 1.32)	0.652	
Q4 (N = 1778)	4.49 (3.67, 5.51)	< 0.001		4.21 (3.43, 5.17)	< 0.001		1.57 (1.28, 1.94)	< 0.001	
SIRI (Quartile^a^)									
Q1 (N = 1776)	Ref.		< 0.001	Ref.		< 0.001	Ref.		< 0.001
Q2 (N = 1782)	1.17 (0.89, 1.52)	0.261		1.19 (0.91, 1.56)	0.205		1.01 (0.74, 1.38)	0.968	
Q3 (N = 1779)	2.02 (1.59, 2.58)	< 0.001		2.01 (1.58, 2.57)	< 0.001		1.13 (0.84, 1.51)	0.430	
Q4 (N = 1778)	5.79 (4.64, 7.23)	< 0.001		5.52 (4.41, 6.90)	< 0.001		1.64 (1.25, 2.17)	< 0.001	
NLR (Quartile^a^)									
Q1 (N = 1783)	Ref.		< 0.001	Ref.		< 0.001	Ref.		< 0.001
Q2 (N = 1775)	0.86 (0.65, 1.13)	0.280		0.90 (0.68, 1.18)	0.433		0.89 (0.65, 1.24)	0.498	
Q3 (N = 1779)	1.94 (1.53, 2.46)	< 0.001		1.92 (1.51, 2.43)	< 0.001		1.20 (0.91, 1.60)	0.200	
Q4 (N = 1778)	5.50 (4.40, 6.82)	< 0.001		5.14 (4.14, 6.39)	< 0.001		1.62 (1.24, 2.12)	< 0.001	
PLR (Quartile^a^)									
Q1 (N = 1784)	Ref.		< 0.001	Ref.		< 0.001	Ref.		< 0.001
Q2 (N = 1807)	0.86 (0.66, 1.12)	0.265		0.85 (0.65, 1.11)	0.243		0.84 (0.61, 1.18)	0.313	
Q3 (N = 1724)	1.97 (1.57, 2.48)	< 0.001		1.81 (1.44, 2.28)	< 0.001		0.99 (0.73, 1.34)	0.957	
Q4 (N = 1800)	4.64 (3.76, 5.73)	< 0.001		4.04 (3.27, 5.01)	< 0.001		1.67 (1.25, 2.23)	< 0.001	
NLPR (Quartile^a^)									
Q1 (N = 1784)	Ref.		< 0.001	Ref.		< 0.001	Ref.		0.059
Q2 (N = 1807)	1.15 (0.90, 1.46)	0.282		1.18 (0.92, 1.51)	0.202		1.12 (0.84, 1.50)	0.432	
Q3 (N = 1724)	1.74 (1.38, 2.19)	< 0.001		1.71 (1.35, 2.16)	< 0.001		1.39 (1.06, 1.82)	0.018	
Q4 (N = 1800)	4.16 (3.38, 5.13)	< 0.001		3.88 (3.14, 4.80)	< 0.001		1.33 (1.03, 1.71)	0.028	
AISI (Quartile^a^)									
Q1 (N = 1779)	Ref.		< 0.001	Ref.		< 0.001	Ref.		< 0.001
Q2 (N = 1778)	0.95 (0.73, 1.23)	0.696		0.97 (0.75, 1.26)	0.835		1.04 (0.76, 1.43)	0.800	
Q3 (N = 1780)	1.64 (1.30, 2.07)	< 0.001		1.63 (1.29, 2.07)	0.002		1.05 (0.78, 1.41)	0.757	
Q4 (N = 1778)	4.77 (3.87, 5.88)	< 0.001		4.57 (3.70, 5.65)	< 0.001		1.80 (1.37, 2.37)	< 0.001	
RDW (Quartile^a^)									
Q1 (N = 2044)	Ref.		< 0.001	Ref.		< 0.001	Ref.		< 0.001
Q2 (N = 1365)	2.90 (2.12, 3.97)	< 0.001		2.65 (1.94, 3.63)	< 0.001		1.62 (1.13, 2.32)	0.009	
Q3 (N = 1876)	4.78 (3.60, 6.34)	< 0.001		4.05 (3.05, 5.40)	< 0.001		1.80 (1.30, 2.52)	< 0.001	
Q4 (N = 1830)	11.38 (8.69, 14.90)	< 0.001		9.81 (7.47, 12.88)	< 0.001		2.96 (2.10, 4.17)	< 0.001	

Model 1: unadjusted; Model 2: adjusted for age, male, race; Model 3: adjusted for age, male, race, heart rate, respiratory rate, saturation of peripheral oxygen, sequential organ failure assessment score, acute exacerbation of chronic obstructive pulmonary disease, congestive heart failure, malignant cancer, dyslipidemia, acute respiratory failure, acute kidney failure, potassium, aniongap, blood urea nitrogen, glucose, serum creatinine, hematocrit, hemoglobin, mean corpuscular volume, red blood cell, dialysis, vasopressor, invasive mechanical ventilation, coronary artery bypass graft, angiotensin-converting enzyme inhibitors or angiotensin receptor blocker, antiplatelet, statin, non-vitamin K antagonist oral anticoagulant, and Vitamin K antagonists.

a RDW categries: Q1 (x < 13.20), Q2 (13.20 ≤ x < 14.00), Q3 (14.00 ≤ x < 15.50), Q4 (x ≥ 15.50); SII categries: Q1 (x < 548.96), Q2 (548.96 ≤ x < 1022.74),Q3 (1022.74 ≤ x < 2167.45), Q4 (x ≥ 2167.45); SIRI categries: Q1 (x < 1.41), Q2 (1.41 ≤ x < 3.17), Q3 (3.17 ≤ x < 6.99), Q4 (x ≥ 6.99); NLR categries: Q1 (x < 3.93), Q2 (3.93 ≤ x < 6.35), Q3 (6.35 ≤ x < 11.38), Q4 (x ≥ 11.35); PLR categries: Q1 (x < 66.67), Q2 (66.67 ≤ x < 113.48), Q3 (113.48 ≤ x < 217.92), Q4 (x ≥ 217.92); NLPR categries: Q1 (x < 0.02), Q2 (0.03 ≤ x < 0.04), Q3 (0.04 ≤ x < 0.68), Q4 (x ≥ 0.68); AISI categries: Q1 (x < 196.08), Q2 (196.08 ≤ x < 518.40), Q3 (518.40 ≤ x < 1349.60), Q4 (x ≥ 1349.60).

AISI, aggregate index of systemic inflammation; CI, confidence interval; HR, hazard ratio; NLPR, neutrophil to lymphocyte platelet ratio; NLR, neutrophil-lymphocyte ratio; PLR, platelet-lymphocyte ratio; RDW, red blood cell volume distribution width; SII, systemic inflammatory index; SIRI, systemic inflammatory response index.

### Association between inflammatory indicators and 90-day ICU mortality in ICU patients with CHD

Based on **Table 4**, the results of multivariate Cox proportional hazards models after adjusting for baseline characteristics, vital signs, comorbidities, laboratory results, interventions, and medications showed the elevated inflammatory indicators, except for the NLPR, were independent risk factors for ICU mortality in ICU patients with CHD, among which the highest risk was for RDW (first quartile as reference group; second quartile: HR = 1.42, 95% CI: 1.04 - 1.93, *P* = 0.028; third quartile: HR = 1.73, 95% CI: 1.30 - 2.30, *P* < 0.001; fourth quartile: HR = 3.04, 95% CI: 2.27 - 4.08, *P* < 0.001) and AISI (first quartile as reference group; fourth quartile: OR = 1.63, 95% CI: 1.28 - 2.08, *P* < 0.001). Furthermore, the association of each inflammation indicator with 90-day ICU mortality when used as a continuous variable was shown in **Figure 2C**, which implied that the greater the values of SII, NLR, PLR, NLPR, and RDW, the higher 90-day ICU mortality of ICU patients with CHD.

**Table 4:** The association of each inflammatory indicator with 90-day ICU mortality in ICU patients with CHD.

Categories	Model 1			Model 2			Model 3		
	HR (95% CI)	*P*-value	*P* for trend	HR (95% CI)	*P*-value	*P* for trend	HR (95% CI)	*P*-value	*P* for trend
SII									
Q1 (N = 1778)	Ref.		< 0.001	Ref.		< 0.001	Ref.		< 0.001
Q2 (N = 1780)	0.77 (0.62, 0.95)	0.017		0.80 (0.64, 1.00)	0.051		0.93 (0.71, 1.22)	0.601	
Q3 (N = 1779)	1.45 (1.20, 1.76)	< 0.001		1.51 (1.24, 1.84)	< 0.001		1.03 (0.80, 1.32)	0.841	
Q4 (N = 1778)	4.02 (3.37, 4.80)	< 0.001		3.78 (3.15, 4.52)	< 0.001		1.48 (1.17, 1.88)	0.001	
SIRI									
Q1 (N = 1776)	Ref.		< 0.001	Ref.		< 0.001	Ref.		< 0.001
Q2 (N = 1782)	1.17 (0.94, 1.46)	0.168		1.20 (0.96, 1.50)	0.111		1.04 (0.80, 1.36)	0.774	
Q3 (N = 1779)	1.95 (1.59, 2.39)	< 0.001		1.96 (1.59, 2.41)	< 0.001		1.11 (0.86, 1.43)	0.411	
Q4 (N = 1778)	5.28 (4.37, 6.38)	< 0.001		5.11 (4.21, 6.19)	< 0.001		1.54 (1.21, 1.97)	< 0.001	
NLR									
Q1 (N = 1774)	Ref.		< 0.001	Ref.		< 0.001	Ref.		< 0.001
Q2 (N = 1781)	0.85 (0.68, 1.07)	0.157		0.89 (0.71, 1.12)	0.320		0.90 (0.68, 1.17)	0.424	
Q3 (N = 1783)	1.76 (1.44, 2.15)	< 0.001		1.73 (1.42, 2.12)	< 0.001		1.09 (0.85, 1.38)	0.509	
Q4 (N = 1777)	4.80 (4.00, 5.76)	< 0.001		4.46 (3.70, 5.37)	< 0.001		1.40 (1.11, 1.77)	0.004	
PLR									
Q1 (N = 1783)	Ref.		< 0.001	Ref.		< 0.001	Ref.		< 0.001
Q2 (N = 1775)	0.95 (0.76, 1.19)	0.634		0.94 (0.74, 1.18)	0.566		0.95 (0.72, 1.26)	0.728	
Q3 (N = 1779)	2.03 (1.67, 2.48)	< 0.001		1.84 (1.50, 2.25)	< 0.001		0.99 (0.76, 1.29)	0.918	
Q4 (N = 1778)	4.89 (4.06, 5.89)	< 0.001		4.18 (3.46, 5.06)	< 0.001		1.63 (1.26, 2.11)	< 0.001	
NLPR									
Q1 (N = 1784)	Ref.		< 0.001	Ref.		< 0.001	Ref.		0.273
Q2 (N = 1807)	1.10 (0.90, 1.35)	0.363		1.13 (0.92, 1.39)	0.251		1.14 (0.89, 1.45)	0.309	
Q3 (N = 1724)	1.54 (1.26, 2.87)	< 0.001		1.49 (1.22, 1.82)	< 0.001		1.22 (0.97, 1.55)	0.096	
Q4 (N = 1800)	3.69 (3.08, 4.41)	< 0.001		4.38 (2.82, 4.06)	< 0.001		1.23 (0.99, 1.54)	0.067	
AISI									
Q1 (N = 1779)	Ref.		< 0.001	Ref.		< 0.001	Ref.		< 0.001
Q2 (N = 1778)	0.91 (0.73, 1.13)	0.405		0.94 (0.75, 1.17)	0.565		0.95 (0.72, 1.24)	0.685	
Q3 (N = 1780)	1.66 (1.36, 2.02)	< 0.001		1.67 (1.37, 2.05)	0.002		1.04 (0.81, 1.35)	0.738	
Q4 (N = 1778)	4.48 (3.74, 5.37)	< 0.001		4.37 (3.64, 5.26)	< 0.001		1.63 (1.28, 2.08)	< 0.001	
RDW									
Q1 (N = 2044)	Ref.		< 0.001	Ref.		< 0.001	Ref.		< 0.001
Q2 (N = 1365)	2.63 (2.01, 3.44)	< 0.001		2.37 (1.81, 3.11)	< 0.001		1.42 (1.04, 1.93)	0.028	
Q3 (N = 1876)	4.68 (3.68, 5.94)	< 0.001		3.89 (3.05, 4.96)	< 0.001		1.73 (1.30, 2.30)	< 0.001	
Q4 (N = 1830)	11.92 (9.48, 14.98)	< 0.001		10.26 (8.13, 12.94)	< 0.001		3.04 (2.27, 4.08)	< 0.001	

Model 1: unadjusted; Model 2: adjusted for age, male, race; Model 3: adjusted for age, male, race, heart rate, respiratory rate, saturation of peripheral oxygen, sequential organ failure assessment score, acute exacerbation of chronic obstructive pulmonary disease, congestive heart failure, malignant cancer, dyslipidemia, acute respiratory failure, acute kidney failure, potassium, aniongap, blood urea nitrogen, glucose, serum creatinine, hematocrit, hemoglobin, mean corpuscular volume, red blood cell, dialysis, vasopressor, invasive mechanical ventilation, coronary artery bypass graft, angiotensin-converting enzyme inhibitors or angiotensin receptor blocker, antiplatelet, statin, non-vitamin K antagonist oral anticoagulant, and Vitamin K antagonists.

a RDW categries: Q1 (x < 13.20), Q2 (13.20 ≤ x < 14.00), Q3 (14.00 ≤ x < 15.50), Q4 (x ≥ 15.50); SII categries: Q1 (x < 548.96), Q2 (548.96 ≤ x < 1022.74),Q3 (1022.74 ≤ x < 2167.45), Q4 (x ≥ 2167.45); SIRI categries: Q1 (x < 1.41), Q2 (1.41 ≤ x < 3.17), Q3 (3.17 ≤ x < 6.99), Q4 (x ≥ 6.99); NLR categries: Q1 (x < 3.93), Q2 (3.93 ≤ x < 6.35), Q3 (6.35 ≤ x < 11.38), Q4 (x ≥ 11.35); PLR categries: Q1 (x < 66.67), Q2 (66.67 ≤ x < 113.48), Q3 (113.48 ≤ x < 217.92), Q4 (x ≥ 217.92); NLPR categries: Q1 (x < 0.02), Q2 (0.03 ≤ x < 0.04), Q3 (0.04 ≤ x < 0.68), Q4 (x ≥ 0.68); AISI categries: Q1 (x < 196.08), Q2 (196.08 ≤ x < 518.40), Q3 (518.40 ≤ x < 1349.60), Q4 (x ≥ 1349.60).

AISI, aggregate index of systemic inflammation; CI, confidence interval; HR, hazard ratio; NLPR, neutrophil to lymphocyte platelet ratio; NLR, neutrophil-lymphocyte ratio; PLR, platelet-lymphocyte ratio; RDW, red blood cell volume distribution width; SII, systemic inflammatory index; SIRI, systemic inflammatory response index.

### Cumulative outcomes based on quartile groups of inflammation indicators

**Figure 3:**
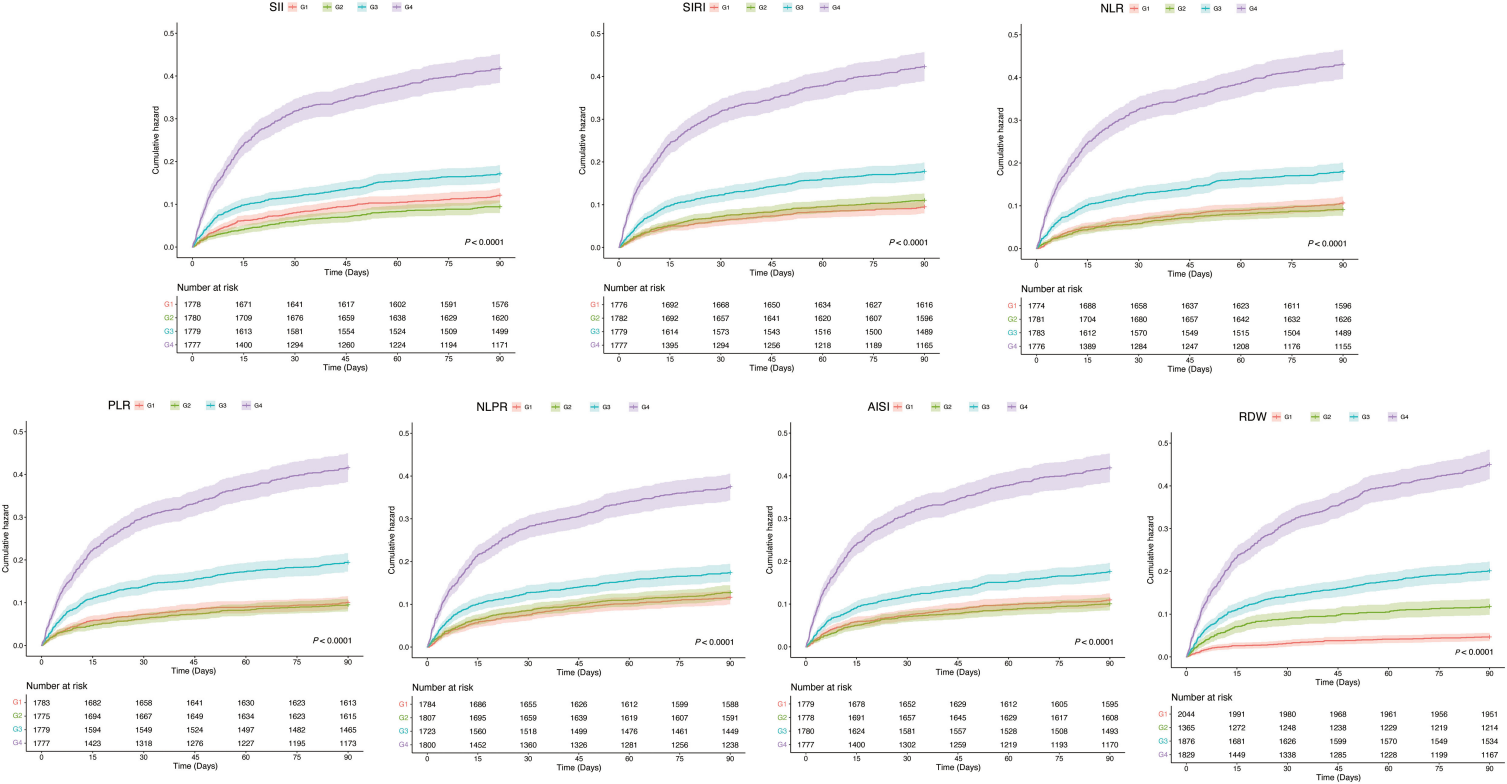
Kaplan-Mill survival analysis of cumulative all-cause mortality in CHD patients within 90 Days of ICU admission. CHD, coronary heart disease; ICU, intensive care unit.

To further explore the association of inflammation indicators with ICU deaths, we plotted Kaplan-Meier cumulative curves for the study outcomes. According to **Figure 3**, among all the inflammation indicators, the higher subgroups had the most cumulative ICU deaths and were statistically different (all *P* < 0.001).

### Development of the novel predictive model

Given that these inflammatory indicators were independent risk factors for ICU mortality in ICU patients with CHD, our aim was to construct a novel model to predict ICU mortality in ICU patients with CHD using these inflammatory indicators combined with the SOFA score. We divided patients from MIMIC into a training cohort (n = 5692) and an internal validation cohort (n = 1423) in an 8:2 ratio. **Table 5** showed the results of univariate logistic regression analyses within the training cohort.

**Table 5:** Univariate and multifactorial analyses about the association between variables and ICU mortality.

Variables	Univariate Analysis		Multivariate Analysis	
	OR (95% CI)	*P*-value	OR (95% CI)	*P*-value
SOFA	1.386 (1.349-1.424)	< 0.001	1.363 (1.325-1.401)	< 0.001
Elevated SII	3.551 (2.924-4.314)	< 0.001		
Elevated SIRI	4.033 (3.318-4.901)	< 0.001		
Elevated NLR	4.069 (3.348-4.945)	< 0.001		
Elevated PLR	2.969 (2.445-3.607)	< 0.001	2.086 (1.631-2.668)	< 0.001
Elevated NLPR	3.212 (2.644-3.901)	< 0.001		
Elevated AISI	3.646 (3.001-4.429)	< 0.001	1.951 (1.535-2.480)	< 0.001
Elevated RDW	3.368 (2.773-4.089)	< 0.001	1.977 (1.586-2.464)	< 0.001

Elevated SII, SII ≥ 2167.45; SIRI, SIRI ≥ 6.99; NLR, NLR ≥ 11.34; PLR, PLR ≥ 217.92; NLPR, NLPR ≥ 0.068; AISI, AISI ≥ 1349.60; RDW, RDW ≥ 15.5.

AISI, aggregate index of systemic inflammation; CI, confidence interval; NLPR, neutrophil to lymphocyte platelet ratio; NLR, neutrophil-lymphocyte ratio; OR, odds ratio; PLR, platelet-lymphocyte ratio; RDW, red blood cell volume distribution width; SII, systemic inflammatory index; SIRI, systemic inflammatory response index.

**Figure 4:**
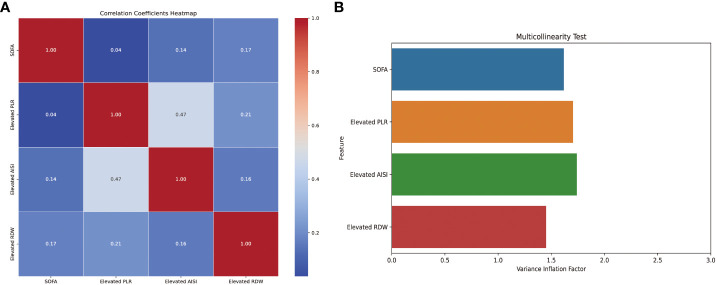
Correlation coefficients and variance inflation factors of the variables in the model. AISI, aggregate index of systemic inflammation; PLR, platelet-lymphocyte ratio; RDW, red blood cell volume distribution width; SOFA, sequential organ failure assessment.

**Figure 5:**
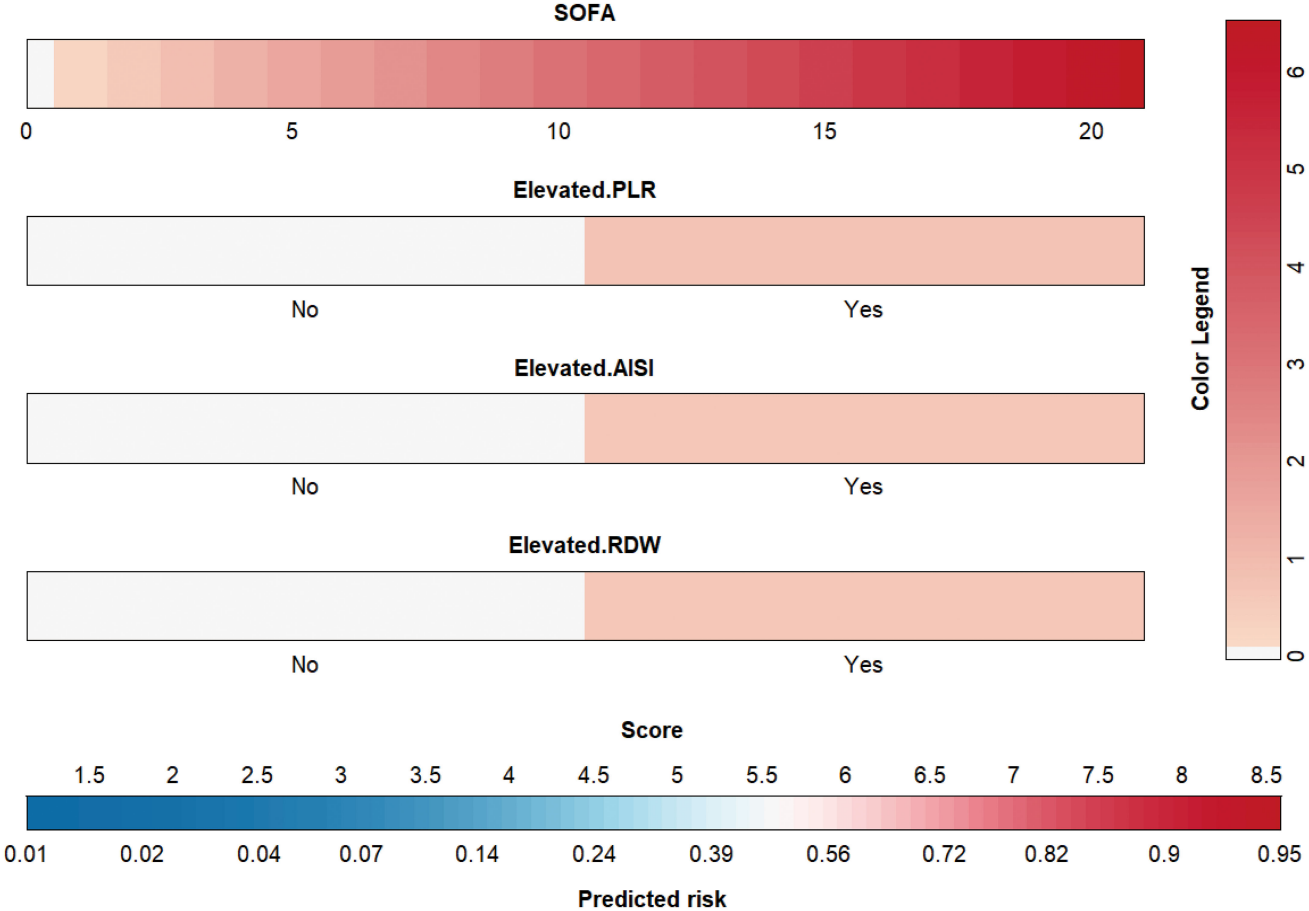
Nomogram for the novel model. AISI, aggregate index of systemic inflammation; PLR, platelet-lymphocyte ratio; RDW, red blood cell volume distribution width; SOFA, sequential organ failure assessment.

Then, based on the results of univariate logistic regression, these variables were subjected to multivariate stepwise forward logistic regression (**Table 5**), and four variables were included in the final predictive model. Regarding the covariance of the variables, correlation coefficient and VIF were calculated and visualized in **Figure 4**, with the results of all variables’ correlation coefficient < 0.5 and VIF < 2, indicating that there was no covariance in the model. The *P*-value of the Hosmer-Lemeshow test was 0.425, indicating that the model was well fitted, and a nomogram was also plotted based on our model (**Figure 5**).

### Validation of the novel predictive model

**Figure 6:**
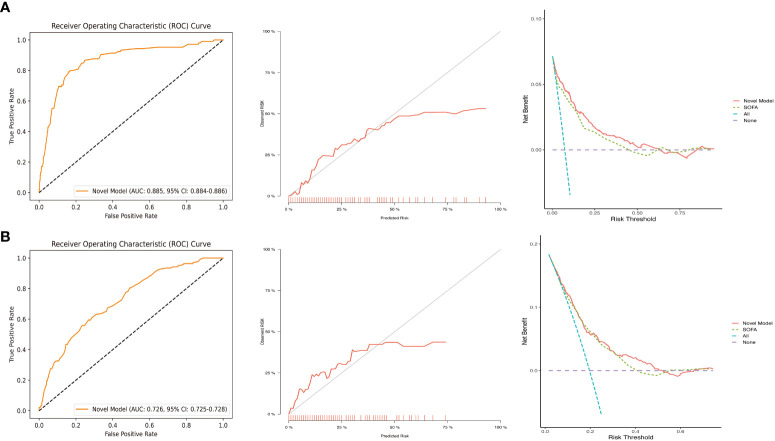
ROC, calibration curves, and DCA in the internal validation cohort **(A)** and external validation cohort **(B)**. DCA, decision curve analysis; ROC, receiver operating characteristic; SOFA, sequential organ failure assessment.

Our external validation cohort was derived from the EICU database, based on the same inclusion and exclusion criteria, the SOFA value was calculated, and excluded individuals with missing data, finally 701 patients were obtained. According to **Figure 6**, the ROC indicated that our model had excellent discriminative performance in both the internal cohort (AUC = 0.885, 95% CI: 0.884 - 0.886) and external validation cohort (AUC = 0.726, 95% CI: 0.725 - 0.728). Furthermore, we used bootstrapping to describe the calibration curves (**Figure 6**), and in both the internal and external validation cohorts, although both the apparent curves and the bias-correction curves deviated slightly from the reference line, the good consistency between observations and predictions were presented.

### Clinical application of the novel predictive model

**Figure 7:**
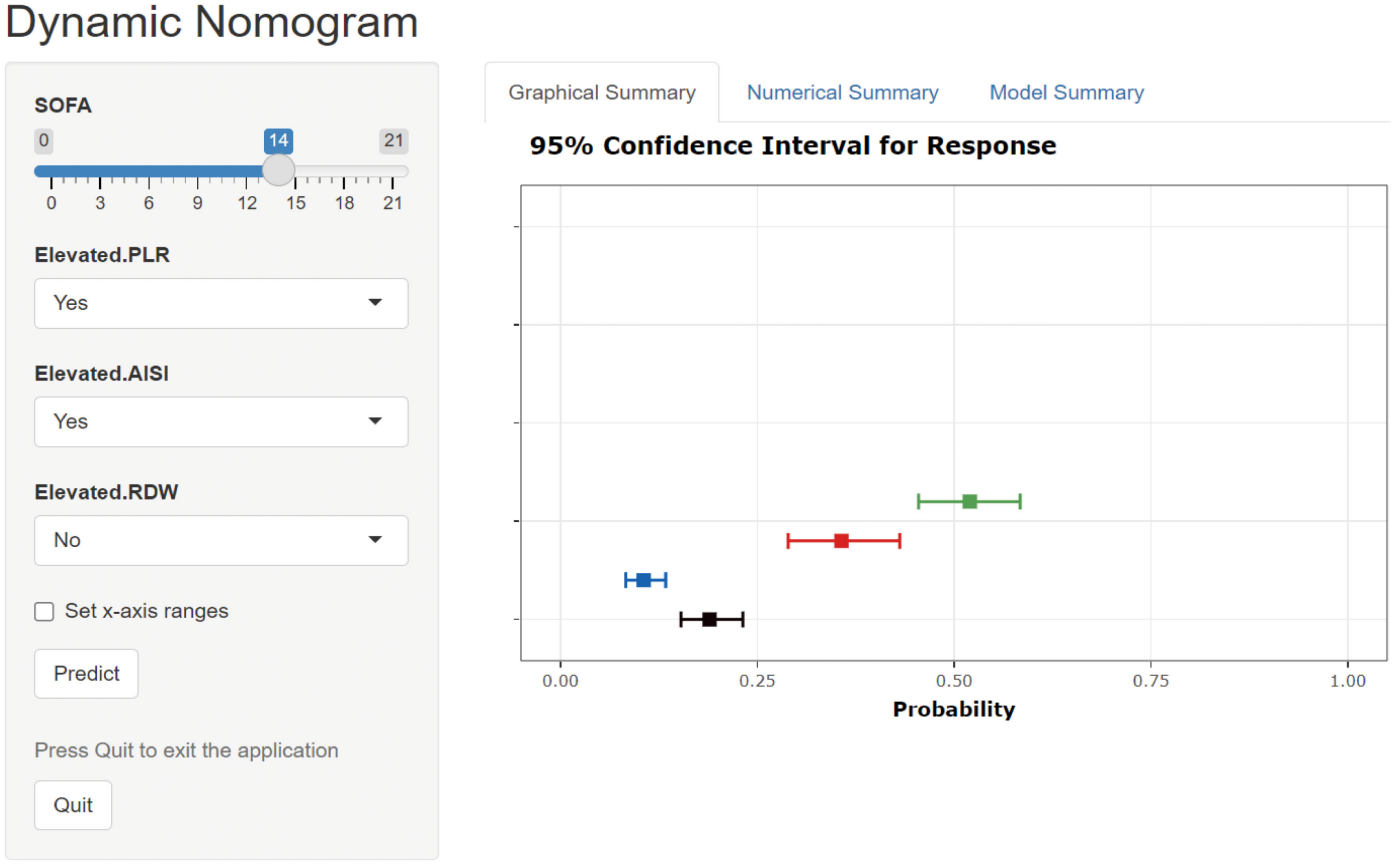
User interface for a novel model-based online prediction tool. AISI, aggregate index of systemic inflammation; PLR, platelet-lymphocyte ratio; RDW, red blood cell volume distribution width; SOFA, sequential organ failure assessment.

To validate the performance of our model for clinical applications, we plotted DCA curves and compared our model to SOFA scores. In both the internal validation set and the external validation set, our model-guided medical interventions provide excellent net benefits and outperform the SOFA score, the details of which are depicted in **Figure 6**. We also compared the IDI difference between our model and the SOFA score, with our model’s predictive performance improving in both the internal and external validation cohorts (internal validation cohort: 6.35% [3.62% - 9.09%]; external validation cohort: 2.12% [0.87% - 3.36%]; both *P* < 0.001). In addition, to facilitate clinical use, we transformed the model into an online dynamic prediction tool with a simple, easy-to-use user interface (**Figure 7**), which is available at https://yangprediction.shinyapps.io/Prediction/.

## Discussion

This was the first study to assess the relationship between the composite inflammation indicators and mortality in ICU patients with CHD. Most inflammation indicators in our study were significantly associated with ICU mortality in the retrospective MIMIC IV database of ICU patients with CHD. Demonstrating the significant promise of these inflammation indicators for mortality risk management in ICU patients with CHD.

The basic mechanism of CHD is abnormal lipid metabolism leading to coronary atherosclerosis (20). Atherosclerosis is commonly characterized by complex interactions between apoptosis and autophagy in endothelial cells, smooth muscle cells, or macrophages, leading to the formation of atherosclerotic plaques (21). Inflammation, which often serves as a risk stratification marker and predicts adverse events, plays an equally important role in the development of atherosclerosis formation (22). Inflammatory-endothelial dysfunction promotes atherosclerosis (23). Of the traditional indicators of inflammation, such as elevated IL-6 may promote elevated CRP levels in those at risk for CHD (24). In circulatory disorders, elevated CRP levels in patients with coronary syndromes, which may progress to heart failure, are strongly correlated with dense plaque composition (25).

Neutrophils, the most abundant subtype of leukocytes in the blood, are critical in mediating inflammation. Neutrophils cause smooth muscle cell lysis and death, which has been shown to cause tissue damage and inflammation in advanced stages of atherosclerosis (26). Platelets actively promote the process of atherosclerosis in response to the interaction of endothelial cells, leukocytes, and unactivated platelets (27). Neutrophil-platelet interaction is an important biological process associated with atherosclerosis, thrombosis, and ischemic stroke (28). Lymphocyte apoptosis tends to increase progressively with increasing atherosclerosis, and lymphocytes are involved in anti-inflammatory and endothelial protection (29). Li et al. evaluated the prognostic values of five lymphocyte-based inflammation indices, including PLR, NLR, SII, and SIRI, in patients with acute coronary syndrome who underwent first-time percutaneous coronary intervention, and found that the addition of NLR, SII, or SIRI, and especially SIRI, to the Global Registry of Acute Coronary Events (GRACE) score, resulted in a better risk-predicting performance than the use of the GRACE score alone (30). It has also been confirmed that NLR is associated with the incidence and susceptibility to carotid plaque detected by carotid ultrasound in patients with acute ischemic stroke (31). And SII was initially defined as the prognosis for cancer, cerebral hemorrhage, and coronary artery stenosis (32). In a previous study, Qu et al. indicated that a lower peripheral hemoglobin to erythrocyte distribution width ratio (HRR) was associated with an increased risk of CHD (33). Decreased HRR is not only associated with decreased hemoglobin, but also with increased RDW.

In our study, RDW was the prognostic independent factor with the most pronounced correlation among the inflammatory indicators studied above. It has been shown that RDW affects the poor prognosis of CHD patients, but the underlying causes remain unclear. Although anaemia is a known risk factor for mortality, we adjusted for haemoglobin in Model 3 and, therefore, still consider RDW to be a risk factor for mortality separate from anaemia. We hypothesized two main causes for the higher risk mortality of CHD patients with high RDW: (a) RDW is associated with a variety of inflammatory markers, e.g., interleukin-6 (34), and erythrocyte sedimentation rate (35). (b) Chronic inflammation may induce disturbances in iron metabolism (36), and the latter is significantly associated with all-cause mortality in CHD patients (37). In addition, RDW may affect dysglycaemia, vitamin D3 deficiency, oxidative stress, and renal failure, all of which are common risk factors for mortality (38).

Our novel predictive model provides an accurate risk assessment and helps ICU physicians identify which ICU patients with CHD are at high mortality risk. And allows ICU healthcare teams to improve the prognosis of high mortality risk patients through more frequent monitoring, more urgent interventions, and stricter medication management at the early stage. In addition, earlier identification of high mortality risk patients can facilitate more informed discussions about the patient’s condition and prognosis between ICU physicians and the patient’s families. Overall, our model has a potentially important role in mortality risk management of ICU patients with CHD, helping to improve the accuracy of clinical decision-making and the quality of healthcare. However, the validity and feasibility of the model have only been confirmed in the datasets. Despite the strong clinical utility of the nomogram, further validation and adjustment is needed in the actual ICU clinical setting.

Some limitations of our study should be noted. First, this was a retrospective study in which retrospective bias is unavoidable, and thus more rigorous prospective studies would be required in the future. Second, previously mentioned traditional inflammatory indicators of IL-6 and CRP are important in mortality risk of CHD. We tried to extract them but due to the limitations of MIMIC IV, the percentage of missing values for CRP was about 98% and no record of IL-6 was retrieved. Further studies are still needed to prove whether they will have an impact on our results. Third, our study data were from the database of the United States and were overwhelmingly of White race, so applicability to ICU patients from other countries or other races requires further validation. Fourth, despite we adjusted for virous potential confounders, there may still be some important factors that were missed, and these may have a non-negligible impact on our inflammatory indicators. For example, neutrophil counts are usually elevated when there is an active infection or inflammation in the patient’s body, while lymphocyte counts may be decreased, thus affecting our inflammatory indicators. However, due to MIMIC’s limitations, it is hard to definitively determine the patient’s infection condition. Fifth, although our novel model demonstrated promising predictive performance in the internal and external validation cohort form MIMIC IV and EICU, however scalability to other hospitals remains an issue as the model’s performance in the external validation cohort is weaker than the internal validation cohort. Therefore, we also need a larger external validation cohort to validate the performance of our model. Despite these limitations, our study shows that the new model we constructed is remarkably promising and deserves further exploration in future clinical work and research.

## Conclusion

Our study revealed that inflammation indicators SII, SIRI, NLR, PLR, NLPR, AISI, and RDW were significantly associated with ICU mortality. Furthermore, we constructed a novel predictive model by combining some of these indicators with SOFA to predict the risk of ICU death in ICU patients with CHD, which has a remarkable potential to guide clinical decision-making and prognostic management.

## Data availability statement

Publicly available datasets were analyzed in this study. This data can be found here: MIMIC IV, EICU.

## Ethics statement

Ethical approval was not required for the study involving humans in accordance with the local legislation and institutional requirements. Written informed consent to participate in this study was not required from the participants or the participants’ legal guardians/next of kin in accordance with the national legislation and the institutional requirements.

## Author contributions

YCheng: Investigation, Methodology, Writing – original draft, Writing – review & editing. YChen: Data curation, Formal Analysis, Investigation, Methodology, Software, Validation, Visualization, Writing – review & editing. MM: Formal Analysis, Software, Writing – original draft. RW: Formal Analysis, Software, Writing – original draft. JZ: Software, Writing – original draft. QH: Methodology, Project administration, Resources, Supervision, Writing – review & editing.
